# Supplementary material for: Content-rich biological network constructed by mining PubMed abstracts
Source: BMC Bioinformatics. 2004 Oct 8;5:147. doi: 10.1186/1471-2105-5-147 (PMC528731; doi:10.1186/1471-2105-5-147)
Supplement: Additional File 5 — The original Chilibot query results of the term "long-term potentiation (LTP)" and 22 other terms, limiting the latest references analyzed to the years 1990, 1995, 2000, and 2004. [file 1471-2105-5-147-S5.bz2 › chilibotAdditionalFile5/ltp2004/html/SYNAPSIN I_CAMKII.html]

 


 **SYNAPSIN I** and **CAMKII** 
  
Found 27 abstracts in PubMed,  **27 abstracts were retrieved and analyzed**.  


---

 Search Google  |
 PDF files only 
|  EDU domain only 

---

**Interactive relationship** (e.g. stimulation, inhibition, etc)

**Parallel relationship** (e.g. studied together, co-existance, homology, etc.)

- This bundling activity is reduced when  **synapsin I**  is phosphorylated by cAMP dependent protein kinase and virtually abolished when it is phosphorylated by calcium calmodulin dependent protein kinase II  [ **CAMKII** ]  or by both kinases.  Ref: 3104800 NatureNature, 1994
- NGF significantly increased the depolarization induced activation of calcium calmodulin dependent protein kinase II  **CaMKII**  and the subsequent phosphorylation of  **synapsin I**  in the absence of external calcium and the NGF effect on evoked glutamate release was inhibited by the  **CaMKII**  inhibitors KN 93 and  **CaMKII**  281 309 peptide but not by the MAP kinase inhibitor PD 98059.  Ref: 12696558 Neuropharmacology, 2003
- After the establishment of synaptic contacts, the presynaptic pool of the kinase displays an increasing level of activity and acquires the parallel ability to phosphorylate  **synapsin I** , which represents one of the major  **CaMKII**  presynaptic targets in mature nerve terminals.  Ref: 12177199 J Neurosci, 2002
- A  **synapsin I**  like protein and calcium calmodulin dependent protein kinase II  [ **CAMKII** ]  were demonstrated by biochemical and immunochemical techniques to be present in squid nervous tissue.  Ref: 2859595 Proc Natl Acad Sci U S A, 1985
- **Synapsin I** , phosphorylated at sites 2 and 3 by purified calcium calmodulin dependent protein kinase II  [ **CAMKII** ] , bound with a 5 fold lower affinity to the vesicles than did dephospho  **synapsin I** .  Ref: 3087973 J Biol Chem, 1986
- phosphorylation of  **synapsin I**  on sites 2 and 3 by calcium calmodulin dependent protein kinase II  [ **CAMKII** ]  removes this inhibitory effect.  Ref: 2512374 J Neurosci, 1989
- **Synapsin I**  and calcium calmodulin dependent protein kinase II  [ **CAMKII** ]  were pressure injected into the preterminal digit of the squid giant synapse to test directly the possible regulation of neurotransmitter release by these substances.  Ref: 2859595 Proc Natl Acad Sci U S A, 1985
- Thus, the sequences surrounding the four sites that are phosphorylated by calcium calmodulin dependent protein kinase II  [ **CAMKII** ] , namely sites 2 and 3 in rat and bovine  **synapsin I** , exhibit a high degree of homology.  Ref: 3118371 Proc Natl Acad Sci U S A, 1987
- METHODS The amounts of DARPP 32,  **synapsin I** , and the alpha subunit of calcium calmodulin dependent protein kinase II  [ **CAMKII** ]  were measured by immunoblotting in postmortem samples from 14 schizophrenic subjects and their age, gender, and autolysis time matched control subjects.  Ref: 12150646 Arch Gen Psychiatry, 2002
- Conversely, injection of calcium calmodulin dependent protein kinase II  [ **CAMKII** ] , which phosphorylates  **synapsin I**  on site II, increased the rate of rise and amplitude and decreased the latency of the postsynaptic potential.  Ref: 2859595 Proc Natl Acad Sci U S A, 1985
- Nervous system tissue from Panulirus interruptus has an enzyme activity that behaves like calcium calmodulin dependent protein kinase II  [ **CAMKII** ]  CaM KII This activity phosphorylates known targets of CaM KII, such as  **synapsin I**  and autocamtide 3.  Ref: 10212401 Invert Neurosci, 1998
- METHODS We investigated the effect of S adenosylmethionine SAM, a compound with putative antidepressant activity, on presynaptic  **CaMKII**  and its synaptic vesicle substrate  **synapsin I** .  Ref: 11543736 Biol Psychiatry, 2001
- Among the several stimulants tested in PC12 cells, bradykinin evoked the phosphorylation of Ser without inducing the autophosphorylation of  **CaMKII** , which was determined using phosphorylation site specific antibodies against phospho Ser  **synapsin I**  and phospho Thr  **CaMKII** .  Ref: 12237306 J Biol Chem, 2002
- The present study presents evidence, using lesion induced degenerations of pre and postsynaptic neuronal populations in the neostriatum and substantia nigra, that calcium calmodulin dependent protein kinase II  [ **CAMKII** ] , as measured both by autophosphorylation of enzyme subunits and by  **synapsin I**  kinase activity, is present in high concentrations in several populations of presynaptic terminals.  Ref: 2545012 Synapse, 1989
- These included several forms of  **synapsin I** , calcium calmodulin dependent protein kinase II  [ **CAMKII** ]  CaM kinase II and avidin.  Ref: 1676419 J Physiol, 1991
- The level of calmodulin and the phosphorylation of  **synapsin I**  in the synaptosomes were not altered up to the 7th day, but the levels of calcium calmodulin dependent protein kinase II  [ **CAMKII** ]  and  **synapsin I**  in the synaptosomes were significantly decreased by microsphere embolism.  Ref: 9507151 Brain Res, 1998
- The ratios of 2 other synaptic phosphoproteins,  **synapsin I**  and the alpha subunit of calcium calmodulin dependent protein kinase II  [ **CAMKII** ] , did not differ between schizophrenic and control subjects, nor between subjects with Alzheimer disease who were and were not treated with neuroleptic agents.  Ref: 12150646 Arch Gen Psychiatry, 2002
- These results suggest that activation of  **CaMKII**  and the concomitant phosphorylation of  **synapsin I**  induce insulin exocytosis from pancreatic beta cells.  Ref: 14501148 J Pharmacol Sci, 2003
- No changes for both  **CaMKII**  and  **synapsin I**  level were found in homogenates, suggesting that synaptic protein changes are not explained by an increase in total level of proteins, but rather by translocation to nerve terminals.  Ref: 11543736 Biol Psychiatry, 2001
- we propose that calcium entry into the nerve terminal activates calcium calmodulin dependent protein kinase II  [ **CAMKII** ] , which phosphorylates  **synapsin I**  on site II, dissociating it from the vesicles and thereby removing a constraint in the release process.  Ref: 2859595 Proc Natl Acad Sci U S A, 1985
- Similar to typical antidepressant drugs, SAM induces changes in  **CaMKII**  activity and increases  **synapsin I**  level in HI and FCX nerve terminals, suggesting a modulatory action on transmitter release.  Ref: 11543736 Biol Psychiatry, 2001
- Immunoblot analysis with two antibodies against  **synapsin I**  phosphorylated by  **CaMKII**  demonstrated the increase in phosphorylation of  **synapsin I**  by the secretagogues.  Ref: 14501148 J Pharmacol Sci, 2003
- Effects of  **synapsin I**  and calcium calmodulin dependent protein kinase II  [ **CAMKII** ]  on spontaneous neurotransmitter release in the squid giant synapse.  Ref: 1978321 Proc Natl Acad Sci U S A, 1990
- Brief freezing of synaptosomes in the presence of gamma 32P ATP and either the catalytic subunit of cyclic AMP dependent protein kinase or calcium calmodulin dependent protein kinase II  [ **CAMKII** ]  rendered the synaptosomal interior accessible to these agents, as reflected by the phosphorylation of substrate proteins, such as  **synapsin I** , which reside within the nerve terminal.  Ref: 2536075 J Neurochem, 1989
- Intraterminal injection of  **synapsin I**  or calcium calmodulin dependent protein kinase II  [ **CAMKII** ]  alters neurotransmitter release at the squid giant synapse.  Ref: 2859595 Proc Natl Acad Sci U S A, 1985
- In order to clarify whether phosphorylation of  **synapsin I**  by  **CaMKII**  was involved in insulin exocytosis, we cloned the isoforms of  **CaMKII**  and  **synapsin I**  from mouse insulinoma MIN6 cells.  Ref: 14501148 J Pharmacol Sci, 2003
- Among the protein molecules specifically located in presynaptic terminals,  **synapsin I**  and calcium calmodulin dependent protein kinase II  [ **CAMKII** ]  CaM kinase II have been shown to modulate evoked transmitter release in the squid giant synapse.  Ref: 1978321 Proc Natl Acad Sci U S A, 1990
- These results raise the possibility that Ser on  **synapsin I**  is alternatively phosphorylated by PAKs, not only by  **CaMKII** , in neuronal cells in response to some stimulants.  Ref: 12237306 J Biol Chem, 2002
- To investigate the changes induced in presynaptic protein phosphorylation by 5 HT reuptake blockade and concomitant  **CaMKII**  up regulation, we analyzed two major  **CaMKII**  presynaptic substrates  **synapsin I**  and synaptotagmin.  Ref: 9016342 Mol Pharmacol, 1997
